# Is the Plague Contagious?

**Published:** 1897-08-07

**Authors:** 


### EDITOR'S LETTER-BOX.

[Our Correspondents are reminded that prolixity is a great bar to publication, and that brevity of style and conciseness of statement greatly facilitate early insertion.]

### IS THE PLAGUE CONTAGIOUS?

Dr. Edward Haughton writes: The gravity of the present situation in India must be my excuse for troubling you with this communication. It has been assumed rather hastily that the intense distrust of the so-called "sanitary measures" which have been forced upon the inhabitants of Poona and other localities where the plague has broken out is altogether unreasonable and may be safely disregarded. There are, however, some facts which may help us to arrive at a somewhat different conclusion. The first is that measures have been carried out in "segregating" healthy people on the bare suspicion of possible infection, which under no circumstances whatever would be tolerated at home, and which may well be supposed to be specially offensive to both Hindoos and Mahometans, who have joined in making a dignified protest acknowledging their willingness to submit to the most stringent regulations if the feelings of the people are respected. If, then, it is possible to do what is really necessary without causing offence one of the principal difficulties of government will disappear and the benefits of real hygiene will become apparent. I have no desire to criticise the details of administration, or even to inquire whether or not some officials may have exceeded their duty. I confine myself to the question, whether it has been proved that the plague is usually propagated by contagion and whether healthy people should be dragged from their homes and prevented from attending to their usual duties and looking after their necessary business under pretence of protecting the country from imaginary microbes. If these microbes were, indeed, capable of multiplying indefinitely it is plain that every epidemic would go on for ever, as their numbers must constantly increase, no matter what measures were adopted to prevent or obstruct human intercourse—even if the doctors were themselves segregated as the persons most likely to convey infection. It is quite certain, however, that epidemics not only die out, but that they tend to become less virulent during that process; even half-buried bodies may abound on every side, and the circle of supposed infectiousness continually increase in area. I know it will be said that we must not judge by isolated cases, yet these have their significance and call for explanation. We see it openly stated that the army of sanitary officials—God save the mark—expect to have another full year of innings in Poona, Bombay, &c. It is impossible to avoid the conclusion that they "think it an ill wind that blows nobody good," and are capable of re-enacting the outrages which have already caused so much discontent in India, provided a sufficient military force is told off "to maintain order." As it is too soon to expect true accounts from India when so many classes of men are interested in concealing them, I now annex some perfectly dispassionate statements which may possibly modify preconceived opinions. In the "Rappor addressé au Conseil de Santé du Caire, 1841." Document 11—"Dr. Ibrahim, a physician at Cairo, states that he was called to see the wife of Hassan Pasha in 1841, whom he found labouring under all the true and severe symptoms of plague. She died at the end of 31 days. This lady had in her service 12 white and 12 black slaves, four eunuchs, and four pages. These were in constant communication with the patient and with the rest of the household of the palace—a hundred persons in all—and yet not one was attacked." We are also informed by Dr. Delong, another physician at Cairo, that a girl, five years of age, in the house of Saad Pinto in the Jews' quarter was attacked with plague in 1841. "She was nursed constantly by her mother; she was surrounded by her brothers, her sisters, and her cousins; and she was in contact with all the household. The young patient died, but all the family continued healthy." The same writer adds, "Two young Turks belonging to the Cadi, the chief judge in Cairo, were attacked with plague about the same time and were placed in the same room. All the numerous persons attached to the palace went to visit them. The visitors took the patients' hands, consoled them, nursed them, and touched them without taking any precautions. The two patients died, one shortly before the other. No other case was observed in all the vast enclosure of the Palace of Justice."—Document 19. I need not say how favourably the behaviour of these unbelievers contrasts with the ideal of Christianity which is now popular, wherein it becomes a moral duty to "pass by on the other side." I conclude with a quotation from Dr. Clob Bey, whose professional position was second to none in the country, and who thus sums up his experience: "I repeat, it has been demonstrated to me by a profound study of the history of this disease, and especially by the numerous facts which I have myself observed, or which have been made known to me by all the medical men who have had to combat the plague in Egypt lately, that the plague is never propagated by contagion." He further adds, "The contact with an infected person is not only not necessary for the production of plague, but it is of itself absolutely innocuous, and is only productive of danger because it brings the individual near the focus of the disease, or of the cause productive of it."—"Reponse sur questions posées par le Ministere Anglais en 1839."

